# “What will the doctor give me, the same painkiller?”: a qualitative study exploring health-care seeking and symptoms self-management among patients for the treatment of long-term chikungunya disease, in Curaçao

**DOI:** 10.1186/s12913-023-10254-8

**Published:** 2023-11-13

**Authors:** Churnalisa Doran, Ashley Duits, Izzy Gerstenbluth, Adriana Tami, Ajay Bailey

**Affiliations:** 1 Department of Medical Microbiology and Infection Prevention, University Medical Center Groningen, Hanzeplein 1, Groningen, 9713 GZ Netherlands; 2 Department of Immunology, Curaçao Biomedical and Health Research Institute, Pater Eeuwensweg 36, Willemstad, Curaçao Curaçao; 3 Department of Epidemiology, Curaçao Biomedical and Health Research Institute, Pater Eeuwensweg 36, Willemstad, Curaçao Curaçao; 4 Department of Human Geography and Spatial Planning, University of Utrecht, Heidelberglaan 8, Utrecht, 3584 CS Netherlands

**Keywords:** Chikungunya, Chronic, Long-term, Health-care seeking, Rheumatic symptoms, Self-management, Treatment, Qualitative

## Abstract

**Background:**

Long-term chikungunya disease, characterized by persistent disabling rheumatic symptoms, including poly-arthralgia/arthritis of severe pain intensity, can persist for years after infection with the re-emerging mosquito-borne chikungunya virus. Although persistent symptoms and pain severity are important determinants of health-care seeking and self-management of symptoms, research on these in relation to long-term chikungunya disease is scarce. This study aimed to explore the perceived benefits and perceived barriers concerning health-care seeking, based on the Health Belief Model, and the symptoms self-management strategies used for health outcome improvement among individuals affected by long-term chikungunya disease.

**Methods:**

An exploratory qualitative descriptive study was conducted with 20 purposively selected adults (17 females and 3 males) with persistent rheumatic symptoms, recruited from an ongoing longitudinal chikungunya cohort, in Curaçao. Semi-structured interviews were carried out, audio-recorded, and transcribed. An iterative coding process was used for themes identification through inductive thematic analyses.

**Results:**

No perceived benefits in health-care seeking were reported. Identified themes in relation to perceived barriers were: (1) health-care seeking at disease onset; (2) general practitioners (GPs) perceptions and awareness of persistent symptoms; (3) challenges for medical referrals and support; (4) no validation of symptoms and challenges accessing therapy; (5) health system restrictions; and (6) social stigmatization of psychological help. These perceived barriers have led participants to self-manage persistent symptoms. Over-the-counter pharmacological and/or non-pharmacological treatments were used without consulting GPs. Identified themes were: (1) self-medication of symptoms; and (2) self-management true non-pharmacological treatments.

**Conclusions:**

To promote the benefits of long-term health-care seeking and subsequently reduce the possible harmful use of analgesics, a collaborative physician-patient therapeutic relationship need to be encouraged. To facilitate this, important shifts may be needed in chikungunya sequalae education of both patients and health-care professionals, and policy makers need to revise health systems for the long-term provision of multidisciplinary care to achieve beneficial health outcomes in long-term chikungunya disease.

**Supplementary Information:**

The online version contains supplementary material available at 10.1186/s12913-023-10254-8.

## Background

Long-term chikungunya disease is defined as persistent disabling musculoskeletal or rheumatic symptoms, lasting > 3 months to years after infection with the re-emerging arboviral chikungunya virus (CHIKV) [1]. Among individuals affected by long-term chikungunya disease, constant or recurrent poly-arthralgia/arthritis (joint pain/joint swelling plus stiffness, respectively) of moderate to severe pain intensity and myalgia, are the most prevalent rheumatic symptoms [2, 3]. In addition, long-term chikungunya disease is associated with inducing non-rheumatic symptoms, including fatigue and emotional distress, severely impairing both physical and psychological health-related quality of life (QoL) [2].

While there have been more than 10 million reported cases of CHIKV infection within the last two decades [4, 5] of which more than 30% may develop long-term disease, due to unknown disease aetiology effective anti-viral vaccines, curative treatments, and disease management guidelines are still lacking [1]. Physical disabilities and pain severity are important determinants of patient health-care seeking and consultations with health-care professionals (HCPs) [6]. However, up to 50% of individuals with severe musculoskeletal pain do not seek medical help, of which many turn to self-medication, with subsequent risk of complications [7]. During the past decades, epidemiologists and social scientists have emphasized that studies regarding health-care seeking will provide good insight and understanding about factors which may have health policy implications for the improvement of health outcomes [8].

The Health Belief Model (HBM) is one of the most widely used conceptual frameworks to study health related behaviours including an individual’s behaviour in response to the presence of symptoms or a diagnosed disease [9]. The HBM proposes that health behaviours are shaped by an individual’s perception or belief of disease susceptibility, severity, benefits and barriers to health practices, self-efficacy, and cues to action [10]. Of all of the HBM components, perceived barriers are the most significant in determining engagement in a behaviour related to change or adaptation for the improvement of health outcomes [11]. For a behaviour to be acted on, an individual needs to believe that the perceived benefits outweigh the perceived barriers [10].

Attaining a good health-care seeking behaviour is a key element of managing long-term disease conditions. However, to date, no attempts have been made to explore the health-care seeking among individuals affected by long-term chikungunya disease, and symptoms self-management strategies used to improve their physical disabilities and QoL. As such, the current qualitative study is the first to focus specifically on the perceived benefits and perceived barriers in relation to health-care seeking and the effectiveness of health-care services rendered by HCPs, and how these experiences and perceptions influence long-term health-care seeking among individuals with long-term chikungunya disease, in an island state in the Caribbean. In addition, this study explored whether symptom self-management strategies were used to relief symptoms and improvement of physical disabilities. Identifying these health-care seeking experiences and perceptions, and self-management strategies could facilitate the design and implementation of disease management interventions to improve the health outcome in long-term chikungunya disease, while curative treatments are being developed.

## Methods

### Study design and setting

The consolidated criteria for reporting qualitative research (COREQ) checklist were used for preparation of the current paper (see Additional File 1) [12]. This study employed a qualitative exploratory descriptive design. Qualitative research methods investigate the perspectives, motivations, beliefs, and needs of individuals in-depth [13]. To ensure rigor in this study, a detailed description of the recruitment process, data collection procedure, and data analysis was provided [14]. The study was conducted in Curaçao, an autonomous country within the kingdom of the Netherlands, located in the southern Caribbean sea. In June-July 2014, CHIKV emerged in Curaçao for the first time, infecting approximately 30–50% of the 150.000 inhabitants [15]. The public health system of Curaçao is similar to the health system of the Netherlands, which is structured into primary, secondary, and tertiary care health sectors. Primary care is provided by general practitioners (GPs), who are the gatekeepers to secondary care [16]. The basic health insurance system provides uniform health-care coverage to all documented inhabitants, executed through the Social Insurance Bank (SVB) [17].

### Patient and public involvement

Our data collection tool was piloted in the developmental phase of the study to assess the length of the interview and ensure that it was inclusive, comprehensive and generated data that met the study objectives. The pilot study was performed through in-depth interviews of 4 individuals affected by long-term chikungunya disease recruited from the general population via snowball sampling. No adjustments were necessary at this stage.

### Participants and recruitment

The participants of this study comprised of adult patients recruited from an ongoing longitudinal chikungunya cohort study with confirmed diagnosis of CHIKV infection, based on acute febrile symptoms and/or a positive laboratory test, during the CHIKV outbreak in Curaçao [15]. The study was approved by the Medical Ethical Committee of the Saint Elisabeth Hospital in Curaçao on the 4th of August 2017 (Reference number: 2017-003). Study participants had to meet the following inclusion criteria: (1) be long-term affected by persistent rheumatic symptoms, since CHIKV infection; (2) have no rheumatic and/or mental or emotional disorders prior to CHIKV infection; and (3) have no fatigue prior to CHIKV infection. In August 2020, eligible participants were selected using cohort records from a follow-up health survey, conducted between July 2019 and March 2020 [18]. During the follow-up health survey, 51 females and 9 males met the inclusion criteria by self-declaring being long-term affected by persistent rheumatic symptoms of various pain intensity, corresponding with reports of more frequent persistent rheumatic symptoms after CHIKV infection in females compared to males [19]. Therefore, for participant selection, maximum variation purposive sampling based on gender, age, and persistent rheumatic symptoms was employed, to allow for a broad range of health-care seeking experiences and symptoms self-management strategies used for the relief of symptoms and improvement of physical disabilities [20].

In September and October 2020, the selected individuals were contacted by phone and invited to participate if they self-declared being still affected by persistent rheumatic symptoms, at time of contact. Out of the 9 males approached, 6 declared being recovered and were excluded. Participant inclusion continued until data saturation was met during data collection. In total 20 individuals were included in the study. All of the contacted individuals agreed to participate.

### Data collection

Data collection took place in September and October 2020. To guarantee both credibility and homogeneity all interviews were conducted by the first author CD, a female researcher with interest in public health, and trained in conducting interviews and fluent in the native local language Papiamentu. Participants were made aware that CD was a PhD candidate at the University Medical Center of Groningen and that she was leading the longitudinal chikungunya cohort study. As an interviewer CD had professional contact with 3 of the included participants during quantitative data collection of the chikungunya cohort [18]. Data were collected in Papiamentu, through a piloted semi-structured topic guide (see Additional File 2) either in the participant’s homes or at work, depending in the participant’s preference. Only CD and the participant were present during the interviews. The topic guide ensured consistency of topics covered and was developed by CD based on a literature review that identified open-ended questions on health-care seeking and symptom management. The topic guide content was evaluated by AB an anthropologist and senior qualitative researcher, to ensure questions aligned with study objectives. The topic guide included the following sections to encourage participants to describe their perceived benefits and perceived barriers in relation to health-care seeking, and symptoms self-management strategies for long-term chikungunya disease: (i) rheumatic and non-rheumatic symptoms present at the time of interview; (ii) health-care seeking for rheumatic and non-rheumatic symptoms; (iii) medical referrals and perceived effectiveness of rendered health-care services; (iv) and symptoms self-management strategies for the relief of symptoms and physical disability. Interviews began with an open question asking the interviewee to describe their symptoms at disease onset and experience of health-care seeking during acute disease; followed by questions relating to each of the four main areas listed above; and ended with an invitation to discuss any topics not already covered.

Each interview lasted between 25 and 75 min and were audio recorded. Field notes were taken through keen observation of the non-verbal cues expressed by participants. Data saturation was reached after 16 interviews, with the final 4 interviews being conducted for confirmation [21]. Saturation was considered to have been met when probing gained no new information between the 15th and 20th interview. No repeat interviews were carried out. The interviews were transcribed verbatim by an independent paid transcriber, native in Papiamentu. Participant’s identifiers were removed from the transcripts. Demographic data related to educational status were extracted from cohort health-survey records.

### Data analyses

The qualitative software ATLAS.ti was utilized for data management, coding, and analysis. The data was analysed using thematic analysis, a method for systematically identifying, analysing, and reporting patterns (themes) of meaning within data [22]. An inductive approach was employed towards the thematic analysis, allowing the codes and themes deriving from the content of the data [23]. The analyses were conducted using the transcripts in Papiamentu. The transcripts were read and re-read by CD to attain pre-analytical insight by listening to the original audio recordings and subsequently checking the transcripts for accuracy. Line-by-line coding in English was used by CD to code individual interview transcripts. Initial codes were refined through constant comparison and modification as each individual transcript was coded and added to the dataset. For quality assurance purpose, AB independently checked two random transcripts translated to English. Subsequently, CD and AB revised the codes, before CD collated the codes into potential themes, until no new themes emerged [22]. The potential themes were discussed between CD and AB for consensus on the final themes. This was conducted to reduce potential subjectivity or researcher bias and ensuring credibility and trustworthiness [22] strengthening dependability. The themes were classified into health-care seeking (see Additional Table 1) and symptoms self-management strategies (see Additional Table 2). Health-care seeking was defined as consulting with a HCP regarding persistent rheumatic and non-rheumatic symptoms with the purpose of receiving medical help either in the form of treatment, referral, or information. Symptom self-management strategies refers to pain treatment and symptom relief using a conventional pharmacological approach and/or non-pharmacological treatments, not prescribed by GPs. Transcripts were not returned to participants to provide feedback on the findings, due to time elapsed between data collection and completion of the analysis. Relevant quotes were selected and translated to English to add further depth to the themes interpreted from the data [22]. Where necessary, quotes were grammatically corrected to improve readability.

## Results

### Study sample

A total of 20 participants, 17 females and 3 males, aged 32 to 77 years with disease duration ranging between 68 and 74 months (6 years) were included in this study. The majority of the participants were in a relationship or married and had an intermediate vocational education or higher. All participants reported persistent rheumatic symptoms in various body locations in the upper extremities (UE) and lower extremities (LE), which were experienced daily, every other day or on a weekly basis. Some participants reported non-rheumatic symptoms including fatigue and emotional distress. The most common reported comorbidity was hypertension. The socio-demographic and clinical characteristics of the participants are presented in Table 1.

**Table 1:** Socio-demographic, and rheumatic and non-rheumatic characteristics of the participants

Characteristics		N (total = 20)
**Gender**		
Female		17
Male		3
**Age range (year)**		
32–49		8
50–65		11
> 65		1
**Relationship status**		
Single/divorced/widowed		7
In relationship/married		11
Unknown		2
**Educational status (highest degree obtained)**		
Primary school or less		2
Secondary school		2
Intermediate vocational school		10
University (of applied sciences)		6
**Employment status**		
Unemployed/homemaker		2
Paid work		16
Retired		2
**Rheumatic symptoms** ^**a**^		
**Arthralgia**		
	Upper extremities	4
	Lower extremities	2
	Both upper and lower extremities	10
**Arthritis (joint swelling and/or stiffness)**		
	Upper extremities	1
	Lower extremities	6
	Both upper and lower extremities	2
**Joint cramps or locking**		
	Upper extremities	2
	Lower extremities	0
	Both upper and lower extremities	1
**Myalgia**		1
**Non-rheumatic symptoms** ^**a**^		
**Fatigue**		4
**Emotional distress** ^**b**^		3
**Comorbidities** ^**a**^		
Cardiovascular disease		3
Hypertension		7
Diabetes mellitus		3
Others^c^		4

^a^More then one answer was possible; ^b^Loss of vitality, sombreness, stress, anxiety, and mood swings; ^c^Anaemia, asthma, and hyperthyroid

### Perceptions and experiences regarding health-care seeking

All participants indicated being informed during acute chikungunya disease onset by their GPs that there is no specific treatment for the rheumatic symptoms and fatigue, and were given symptomatic treatments such as analgesics and anti-inflammatory medications, including non-steroidal anti-inflammatory drugs (NSAIDs) to manage their rheumatic symptoms and improve their physical disabilities. Some were told that the symptoms may persist and others did not receive any information regarding possible disease duration. In relation to long-term health-care seeking when rheumatic symptoms persisted, participants reported perceived barriers and no perceived benefits. Participants’ perceptions and experiences regarding barriers in health-care seeking included: (1) health-care seeking at disease onset; (2) GPs perceptions and awareness of persistent symptoms; (3) challenges for medical referrals and support; (4) no validation of symptoms and challenges accessing therapy; (5) health system restrictions; and (6) social stigmatization of psychological help. Additional Table 1 presents the code list with codes and additional illustrative quotes related to health-care seeking themes.

### Health-care seeking at Disease onset

The first theme that was identified describes participant’s perceptions of having no medical benefit to gain by attending their GPs, since their GPs focused mostly on communicating that if rheumatic symptoms persist they can only be managed and not cured, during health-care seeking at disease onset. Without discussing any possible pain management interventions, participants were also told to learn to live with persistent symptoms, leaving them with no perceived reason to continue seeking care, as one participant described:

*“I am very honest, when I go to the doctor [GP], what will the doctor tell me, what will the doctor tell me? That is what I am thinking, because in the beginning he [doctor] was saying that you just need to live with it [persistent symptoms]…and for me, when a doctor tells me that, what will I continue going to the doctor for?” (Pt. 19; Female 32–49 years old, arthralgia and joint weakness in UE and LE)*.

Referring to their past health-care seeking, participants also mentioned that the only thing that their GPs did to address their rheumatic symptoms was prescribing analgesics during acute disease, which they can buy over-the-counter themselves. Another participant expressed:

*“What did the doctor give you [talking in third person] during that time [at acute disease onset]? Paracetamol? So, now I will buy the paracetamol myself instead of going to the doctor and being ridiculous.” (Pt. 9; Female 32–49 years old, arthralgia and joint weakness in UE, and fatigue)*.

### GPs perceptions and awareness of persistent symptoms

Another unsatisfactory aspect that served as a perceived barrier for long-term health-care seeking was that in some cases participants expressed that the significance or severeness of their rheumatic symptoms was downplayed or not considered as being related to long-term chikungunya disease by GPs. This caused them to disengage in seeking further help. One participant stated:

*“It [chikungunya] affects your joints, my GP told me no [that chikungunya does not affect the joints]…but I have told my GP that since I had that thing [chikungunya infection], I remained with the pains in my joints. […]. Now [at this point in time], you know…I do not go to the GP for that [persistent symptoms], because the last time I went to my GP, I talked to him regarding that, I explained it [persistent symptoms] and asked him if they [persistent symptoms] are the effects of chikungunya, he told me he does not think so. So, I drew the line [discontinued health-care seeking].” (Pt. 7; Female 50–65 years old, arthralgia in UE and LE)*.

In addition, in some cases participants perceived that GPs did not listen to their problems and were not interested in identifying the cause of why their symptoms persisted. One participant expressed how he experienced his complaints were not taken serious by his GP:

*“It [chikungunya] left me a little handicapped. I have cramps in my hand and feet, since I got it [chikungunya]…I still have them [cramps]. I went to the doctor [GP] and he told me…eat bananas, I asked the doctor…Bananas? What can bananas help me with? He told me to…eat bananas, bananas will stop the cramps. I told the doctor…I need to go to a specialist to see why [cramps persistency], maybe I have a clogged vein in my hand.” (Pt. 4; Male 50–65 years old, joint cramps in UE and LE)*.

### Challenges for medical referrals and support

Some of the participants expressed being persistent in insisting a referral for medical checks to find a medical explanation for the persistency of symptoms or treatment that would reduce their symptoms and physical disabilities. However, during consultations with their GPs participants often perceived that their requests for medical checks or medical referrals to secondary care was experienced as a battle or a fight, in which participants had to do their utmost best to convince or beg their GPs to write them a referral. One participant expressed:

*“I think here in Curaçao its very bad…you need to beg to be able to go to a physiotherapist, you need to beg to get a referral to go to a laboratory.” (Pt 1; Female 50–65 years old, arthralgia in LE)*.

Participants also stated that one of their main reasons for not attending their GPs is that GPs tend to have a preference to contain health costs down by medicating instead of writing a referral. Another participant expressed how this fostered a reluctant attitude and demotivation towards health-care seeking, experienced by many participants:

*“When I go to the doctor [GP] he gives me medication [analgesics] and I want to test [referral to secondary care] to see why I have pain, so why go [to GP]?” (Pt. 17; Female 50–65 years old, arthralgia in UE and LE, joint locking in UE and LE, joint cramps in UE, joint swelling in LE, fatigue, and emotional distress)*.

### No validation of symptoms and challenges accessing therapy

Participants that received a referral for medical checks including blood circulation, radiography, and neurology tests, to find a cause or source for the persistence of their symptoms, reported not finding a medical explanation by secondary HCPs for the persistency of their symptoms. This created difficulties in the validation of their symptoms, thereby forming a barrier in health-care seeking. One participant expressed how she felt disheartened:

*“My ankles remained swollen [after CHIKV infection] and my knee is painful, I recently got a radiography [test] to see if there is no problem with the circulation [vascular system], but everything was okay [nothing to be seen in radiographical image], so there is nothing more that I can do.” (Pt. 3; Female 50–65 years old, arthralgia and joint swelling in LE)*.

Others expressed having obtained referrals for physiotherapy sessions to relief pain sensations. However, many were dissatisfied with the service, treatment effectiveness, or unpleasant worsening of the pain sensations during or after therapy. One participant explained how the treatment outcome was not worth the worsening of pain, which subsequently led many participants into stopping with physiotherapy:

*“At first I went to therapy, I went to therapy often. Yes physiotherapy, I went often, but I stopped going, because I began noticing that when I went, I would get more pain than before I went to therapy. […]. Often I had to drink painkillers [to reduce the increased pain], so I said no, I do not want to increase the pain for it to decrease afterwards.” (Pt. 7; Female 50–65 years old, arthralgia in UE and LE)*.

Participants who were unhappy with the physiotherapy service and had the financial means, expressed turning to the private sector to obtain physiotherapy and experienced that the service provided there was much better, as one participant expressed:

*“You know, I resent the physiotherapists here in Curaçao. They will put you on a machine and walk away, for me that isn’t physiotherapy. […]. Physiotherapy is really the contact [manual contact]. […]. I prefer to pay a massage and let them [private sector physiotherapist] do a body massage two times a month, sometimes only shoulders, depending on the wallet [finances]…but I do not go to the therapy, so the medical one [provided by health system].” (Pt. 17; Female 50–65 years old, arthralgia in UE and LE, joint locking in UE and LE, joint cramps in UE, joint swelling in LE, fatigue, and emotional distress)*.

### Health system restrictions

Participants who were satisfied with the physiotherapy treatment outcome and wanted to continue with therapy, blamed the health system for not allowing the required amount of therapy sessions for sustained pain management. As the participants already paid into the health system as taxpayers, they expected sustained health-care services that better serve their needs. One participant expressed her frustration imposed by the restrictions of the health system in the amount of therapy sessions:

*“The good thing of therapy is to get the massage to spread the pains. […]. The doctor [GP] would tell me, this is only what the SVB [Social Insurance Bank] gave you 4 days [times], 5 days [times]. I think that the whole year SVB is GETTING [emphasizing on getting] my money and I do not make use of it, you understand? I am serious, I want them [policy makers] to improve that.” (Pt. 13; Female 50–65 years old, arthralgia and joint stiffness in UE and LE).*

Another participant explained that even HCPs who wanted to continue with sustained pain management are conditioned and restricted by the barriers of the health system:

*“I also think that the therapist [physiotherapist] also thought that he was not allowed to go and asks for more [physiotherapy sessions]…Because those are the rules of the SVB [Social Insurance Bank], so till an amount [sessions] you can receive therapy.” (Pt 10; Female > 65 years old, arthralgia in UE and LE, and joint stiffness and swelling in LE)*.

### Social stigmatization of psychological help

In relation to seeking health-care for emotional distress, most participants described being aware of the benefits of psychological treatment in coping with the mental or emotional distress of living with persistent rheumatic symptoms. However, all participants including the ones that reported emotional distress, stressed that they do not need nor seek psychological care:

*“I think that maybe some people do need it [psychological help], but I do not need it.” (Pt 19; Female 32–49 years old, arthralgia and joint weakness in UE and LE, and emotional distress*.

It was mentioned that seeking psychological care is a perceived barrier in the small island community. Hiding mental or emotional distress is socially and culturally common, because of the fear of being stigmatized or labelled as crazy within the community, being seen as dysfunctional, and/or that psychologists are acquainted with social network and therefore can’t be trusted. One participant suggested that establishing a peer group, may benefit individuals with emotional distress induced by persistent rheumatic symptoms:

*“Why do they [HCPs] not establish an association like the stomach [gastrointestinal] association, but for chikungunya? …because until now there is none, you can establish one and people who have psychological problems can start talking [in a group] and you can even include a psychologist to give talks etc. A place where they [individuals with emotional distress induced by persistent rheumatic symptoms] can come and share their experience to help each other, one helps the other, one supports the other, you know… and in that way you break the barriers.” (Pt. 7; Female 50–65 years old, arthralgia in UE and LE)*.

### Self-management strategies of persistent symptoms

Overall, these aforementioned perceived barriers have led to the perception among participants that they need to self-manage their persistent symptoms independently. Some participants reported that although they still experienced episodes of pain and physical disabilities, it considerably improved compared to during acute disease onset, which made the symptoms easier to cope with and a disincentive for taking analgesics. Themes related to self-management strategies for persistent symptoms included: (1) self-medication of symptoms; and (2) self-management true non-pharmacological treatments. Additional Table 2 presents the code list with codes and additional illustrative quotes related to symptoms self-management strategies themes.

### Self-medication of symptoms

To function and participate in activities of daily living, more than half of the participants used over-the-counter conventional oral analgesics as their preferred strategy for achieving an acceptable level of pain relief and improvement of physical disabilities. Oral NSAIDs like Diclofenac (including brand names Dicloflex-Forte, Dolo-Neurobion, and Voltaren) and Ponstan Forte were used and generally experienced as superior to paracetamol, due their stronger and longer perceived analgesic effect of 1–2 days. As one participant explained:

*“Paracetamol lasts only for a short period. […]. After 2 or 3 hours, you need to repeat the same dosage again. I drink a pill from Santo Domingo [Dominican Republic], which is Diclofenac with vitamins [Dicloflex-Forte]. […]. I will drink [take] it in the morning and till night I will stay good [without pain].” (Pt. 15; Female 32–49 years old, arthralgia and weakness in UE and LE)*.

Even though, paracetamol was generally mentioned as less effective compared to NSAIDs, participants were very cautious and reluctant about taking all oral analgesics, weighing the risks and benefits associating them with potential drug dependency, addiction, and adverse side effects. One participant expressed:

*“Yes it’s [Diclofenac] very strong, but I do not drink it alone, I have another pill that I drink [take] before I drink [take] the Diclofenac…for the stomach, to protect the stomach.” (Pt. 18; Male 50–65 years old, myalgia in UE)*.

On the other hand, many participants often felt having no choice other than taking analgesics every other day or once a week, when their pain was nagging or unbearable, as one participant stated:

*“I do not take painkillers immediately. I will observe how it [pain] is going, if I notice that it’s not going really really [unbearable] I will take painkillers…maybe I will stay one day with the pain and the next day I will say…no this pain has become too much…I will then take a medication [painkiller], because the pain hampers me.” (Pt. 17; Female 50–65 years old, arthralgia in UE and LE, joint locking in UE and LE, joint cramps in UE, joint swelling in LE, fatigue, and emotional distress)*.

Participants have also tried topical NSAIDs analgesics as monotherapy or add-on treatment for obtaining localized pain relief and/or to refrain from taking oral NSAIDs. They mentioned using gels and creams containing Diclofenac. One participant explained:

*“Like at night I will smear the Diclofenac gel, in the morning I will wake up with less pain. I can stand up and go to the bathroom.” (Pt. 6; Female > 65 years old, arthralgia and joint stiffness in LE)*.

Topical analgesics were perceived as having a localized analgesic effect by some, while others did not notice sufficient pain relief, as one participant expressed:

*“I buy things [topical analgesics] to smear on my leg. […]. None of them work, they alleviate it [pain] just a little, they [topical analgesics] do not stop it [pain].” (Pt. 20; Female 50–65 years old, arthralgia and joint cramps in UE, joint stiffness in LE, and emotional distress)*.

### Self-management true non-pharmacological treatments

Different non-pharmacological treatments for reducing tiredness and fatigue, and temporally pain alleviation were implemented, including vitamins, resting and stretching, wrapping of the joints with clothing to keep the joints warm, warm/cold baths with baking soda, and ice packages. One participant described:

*“I have ice packages and at night I will put one here [on the knee], and then one here [on the ankle] and one here [on the shoulder]. […]. I will have less pain for around two days.” (Pt 10; Female > 65 years old, arthralgia in UE and LE, and joint stiffness and swelling in LE)*.

Many participants also used natural alternative remedies including ‘’Green’’ (a menthol gel or cream containing cannabis sativa seed oil), herbal tea and syrup extracts of lemons, ginger, papaya and mango leaves during and after disease onset, yet whether the symptom reduction was caused by possible beneficial effects of the herb extracts or reduced pain intensity over time was questioned. However, despite their high costs, some participants were desperate to try every non-pharmacological treatment that was claimed to be effective, without success, as one participant expressed:

*“You know what I do? I try to go to every natural doctor to find a solution…If you tell me madam go to doctor XYZ, that doctor will help you and it costs 500 Antillean Guilders [$ 278] so to speak, I will save [for the treatment], because I think that now I have found a cure for it [persistent symptoms].[…]. If they [social network] tell me…there is another natural doctor let’s go, I will start my car and go. If they [social network] tell me…let’s go there, I will go…No, let’s go there, I will go. I went to [name natural product store], I have gone everywhere people say that can help.” (Pt. 19; Female 32–49 years old, arthralgia and joint weakness in UE and LE)*.

## Discussion

The current explorative qualitative study aimed to identify the perceived benefits and perceived barriers related to long-term health-care seeking, and symptoms self-management strategies used among individuals with long-term chikungunya disease. No perceived benefits in long-term health-care seeking for persistent rheumatic symptoms were reported. Health-care seeking during acute disease onset, GPs perceptions and lack of symptom awareness, battle in obtaining medical referrals, no medical validation for symptoms persistency, health system restrictions in treatment continuity, and social stigmatization of psychological help were among the perceived barriers to health-care seeking. As a result, participants turned to a variety of over-the counter pharmacological and non-pharmacological treatments, and alternative remedies to control their symptoms and self-manage their physical disabilities, because persistent symptoms have a significant negative effect on their daily lives.

Our results are consistent with those of a previous chronic pain study, in which past health-care seeking experience during and after symptoms onset, and diminished positive health outcome expectancy, were the predominant barriers in seeking health-care when symptoms persisted [7]. Subsequently, participants perceived the management of the symptoms as their own responsibility [24]. This finding has also been identified in other qualitative studies, where chronic pain patients who perceived their health-care provider as unable to help them above their own self-management abilities did not seek long-term health-care [25, 26].

Moreover, our study also revealed that considerable disappointment towards HCPs were experienced, whether for giving priority to the financial considerations imposed by the features of the health system attempting to contain health costs [27], perceived battles to obtain medical referrals [28], or failing to take them seriously by discounting their symptoms. One reason may be that GPs may not be well-trained to manage chronic pain [29]. On the other hand, in addition to the unknown aetiology of chikungunya disease [1], from a medical perspective it is uncommon that an arboviral disease causes long-term rheumatic symptoms, which may persist years after infection [30]. Therefore, without a positive diagnostic test to identify the attribution of the cause of symptoms persistency, discounting symptoms or having a sceptical attitude towards patients by HCPs may be due to lack of knowledge or awareness of chikungunya disease sequalae. Patients with medically unexplained symptoms disengage from health services when they face processes that invalidate their suffering [31].

In addition, none of the participants with emotional distress in this qualitative study indicated being in need of seeking psychological treatment. This study provided a glimpse into the perceived stigmatization to seek psychological care in the social and cultural context of a small island, which need to be addressed. Indeed, a recent study reported a high prevalence of mental health stigmatization in 6 small island developing states, in the Caribbean [32]. Raising the awareness of the public regarding psychological distress and trust in the professionality of psychologist with culturally appropriate education and anti-stigma campaigns, may not only be beneficial in reducing the QoL impairment of those in need including individuals with long-term chikungunya disease, but also informing the public on an important health issue in general [33].

Moreover, beside health-care seeking we were interested whether rendered health-care services benefitted the participants in alleviating symptoms. However, non-adherence to physiotherapy due to increased pain sensations during and after physiotherapy were reported. This result confirms non-adherence in previous rheumatoid arthritis and fibromyalgia studies, were adverse events and/or no perceived benefit of physical therapy was experienced [34–36].

The above mentioned perceived barriers in health-care seeking led to different treatment attempts in search for pain relief and management of symptoms. Our study sample included mainly relatively highly educated individuals, which has been associated with superior health-care literacy and may increase the engagement in symptoms self-management [37]. Participants who were less physically impaired were more likely to seek non-pharmacological therapies [38, 39]. However, most participants continued to use over-the-counter analgesic including NSAIDs, since acute disease onset, without consulting their GPs, but the side-effects and risk/benefit ratio of long-term use and/or high dose of NSAIDs may be underestimated [40]. A major disadvantage of long-term use and/or high doses of NSAIDs are the gastrointestinal side-effects, involvement in renal impairment, and increased risk of cardiovascular disease [41]. In addition, half of our study sample reported having co-morbid conditions, including diabetes and cardiovascular diseases. It is known that comorbidities and co-medication may increase the risk of NSAIDs side-effects [42]. Therefore, individuals with long-term chikungunya should ideally only use NSAIDs after careful case-by-case evaluation of their comorbidities and comedications by their GPs, and to subsequently monitor possible adverse side effects properly in a therapeutic relationship.

The barriers presented above follow the theory of the HBM. The HBM proposes that for a behaviour to be changed or adapted an individual needs to believe that the perceived benefits of health-care seeking outweigh the perceived barriers [10]. The present study has identified a number of perceived barriers amendable to change to improve long-term health-care seeking behaviour. Explanations and patient education concerning the medical knowledge of chikungunya disease sequalae, including its unknown aetiology and lack of diagnostic confirmation tests, are crucial points in the clinical communication between HCPs and individuals affected by long-term chikungunya, because the latter are patients with persistent rheumatic symptoms that cannot be explained medically [43]. To reduce the perceived unwillingness of GPs in guiding symptom management by patients, and subsequent reduction of the possible harmful use of analgesic by those affected, a therapeutic relationship in the clinical encounter preferably at the clinical confirmation of a CHIKV infection need to be encouraged [44, 45]. However, due to the lack of scientific knowledge concerning its aetiology, diagnosis and treatment gaps, HCPs may feel uncertain when dealing with long-term chikungunya disease and consequently to treat their patients [43]. Health-care providers’ decisions about medical referrals and tests, and medication prescription are influenced by their perceptions. Thus, the perception of HCPs will determine the medical care their patients receive [46]. Medical education about the chikungunya disease sequelae, psychological distress, and treatment and rehabilitation options in countries affected by CHIKV outbreaks [47], would increase the knowledge of HCPs and raise awareness of the particular needs of patients to help them cope and improve their health outcomes, and QoL. This is particularly true in case of GPs because they can provide access to secondary health-care.

In addition, to effectively manage both the physical and psychological symptoms of long-term chikungunya disease a multidisciplinary team care approach consisting of primary health-care physicians, rheumatologists, physical therapist, psychologist and peer support groups, which is also the optimal care strategy for individuals with musculoskeletal disorders including rheumatoid arthritis, need to be implemented [48, 49]. Moreover, patients treatment outcome expectations need to be discussed to decrease non-adherence, and the disease management interventions included in the treatment plans need to be long-term accessible for the encouragement of long-term health-care seeking and reduce the harmful use of analgesics by patients. However, there needs to be a shift moving away from an acute health-care model, in which health-care services are provided in an episodic manner when individuals present with symptoms, to a chronic health-care model, in which resources of the health system are better distributed by policy makers to provide consistent high quality health-care over time.

The findings of this study should be viewed in light of some limitations. First, some participants had professional contact with the interviewer through a previously conducted quantitative interview. This could have influenced these participant’s responses. However, the open responses and feedback provided by the participants suggests that this was not a barrier to them sharing their perspectives and experiences. In addition, since all participants agreed to participate, they may have viewed the interviews as necessary and valued having their experiences heard [50], as this may have been the first time participants have been asked about these issues. Moreover, the participants were not clinically examined by a clinician to verify the self-declared symptoms. However, the self-reported symptoms are similar to those reported in studies reporting on long-term sequalae of chikungunya disease [51]. Our study sample also lacked diversity in education level. Another limitation is that recall bias could have occurred particularly on recounting health-care seeking during acute disease onset. In addition, the interview transcripts were not returned to participants to provide feedback on the findings. However, despite the advantages of returning transcripts to interviewees, transcripts transference may cause ethical, methodological and research credibility problems [52]. Furthermore, all participants had access to health-care due to the uniform health coverage system. Hence, our study may not reflect the experiences of individuals who lack access to health-care. However, due to the unknown aetiology and no curative treatment, the insights and experiences especially the symptoms self-management strategies are likely to have transferable commonalities for other long-term affected patients worldwide. Finally, we did not include the GPs perspective as a primary source of information. Future studies should examine their disease knowledge, decision-making process and challenges, and the patient-physician relationship from their perspectives. Notably, during an information session in Curaçao regarding long-term chikungunya disease sequelae with more than 30% of the local GPs, none of the attending GPs reported treating patients for persistent rheumatic symptoms after CHIKV infection.

In conclusion, the findings of this qualitative study identified perceived barriers in long-term health-care seeking and possible harmful use of over-the-counter analgesics for symptom relief, among individuals affected by long-term chikungunya disease. Based on our findings, important shifts may be needed in chikungunya sequalae education of patients and HCPs to encourage a collaborative therapeutic physician-patient relationship in the management of long-term chikungunya disease, and the revision of health systems by policy makers to offer greater support through the long-term provision of multidisciplinary therapy.

### Electronic supplementary material

Below is the link to the electronic supplementary material.

Supplementary Material 1

Supplementary Material 2

Supplementary Material 3

Supplementary Material 4

## Data Availability

The datasets generated and/or analysed during the current study are not publicly available due to participants did not consent to have their full provided information available for the public but are available from the corresponding author on reasonable request.

## Abbreviations

CHIKV: chikungunya virus
COREQ: criteria for reporting qualitative research
GPs: general practitioners
HCPs: health care professionals
QoL: health-related quality of life
LE: lower extremities
NSAIDs: non-steroidal anti-inflammatory drugs
SVB: Social Insurance Bank
UE: upper extremities
