# Supplementary material for: “What will the doctor give me, the same painkiller?”: a qualitative study exploring health-care seeking and symptoms self-management among patients for the treatment of long-term chikungunya disease, in Curaçao
Source: BMC Health Serv Res. 2023 Nov 13;23:1247. doi: 10.1186/s12913-023-10254-8 (PMC10641972; doi:10.1186/s12913-023-10254-8)
Supplement: Supplementary file 2 — Supplementary Material 2 [file 12913_2023_10254_MOESM2_ESM.docx]

**Additional File 2. Interview topic guide.**

1. Could you introduce yourself, what is your age, the amount of people living with you in your household, and occupation?

**Persistent rheumatic and non-rheumatic symptoms, and comorbidities**

1. Would you please describe your experience with chikungunya disease, starting from the first symptoms?
2. What are the symptoms that you are still experiencing, since chikungunya infection?
3. Describe the rheumatic symptoms; joint pain, swelling, stiffness, cramps and/or locking, and body locations.
4. Are the rheumatic symptoms constant or recurrent (come and go)?
5. How long will the recurrent symptoms last?
6. Describe the non-rheumatic symptoms, if you have them: fatigue, sombreness, loss of vitality, stress etc.
7. Do you have any other chronic disease besides long-term chikungunya disease?
   1. How long do you have this disease?

**Health-care seeking for persistent symptoms**

1. What kind of information did your general practitioner gave you regarding the duration of chikungunya disease and its symptoms?
2. Who have you consulted regarding the symptoms that you are still experiencing?
   1. Conventional (general practitioner, pharmacists etc.) and/or alternative health-care provider.
3. When you indicated that you have persistent symptoms to your general practitioner, did he/she relate them to chikungunya disease?
4. How supported do you feel regarding your health concerns when you visit your general practitioner or health-care provider regarding persistent chikungunya symptoms?
5. What are the things that prevent you to seek medical help and what can be done to improve your help seeking?
6. What are your thoughts regarding psychological help for emotional support and have you sought emotional help for persistent chikungunya symptoms?

**Medical referrals to health-care services**

1. To which health-care provider (physiotherapist, psychologist, pain specialist, or other health-care professional) did your general practitioner refer you for persistent chikungunya symptoms?
2. What did your general practitioner or other health-care provider do to address the symptoms or pain and how helpful was it in relieving the symptoms?

**Symptoms self-management strategies**

1. What are your thoughts on conventional (analgesics) and/or non-pharmacological treatments?
2. Which conventional (analgesics) and/or non-pharmacological are you using and what are the benefits and/or disadvantages?

**Ending**

Is there anything else that you would like to say that we may have not discussed or covered already?
