# Supplementary material for: “What will the doctor give me, the same painkiller?”: a qualitative study exploring health-care seeking and symptoms self-management among patients for the treatment of long-term chikungunya disease, in Curaçao
Source: BMC Health Serv Res. 2023 Nov 13;23:1247. doi: 10.1186/s12913-023-10254-8 (PMC10641972; doi:10.1186/s12913-023-10254-8)
Supplement: Supplementary file 3 — Supplementary Material 3 [file 12913_2023_10254_MOESM3_ESM.docx]

**Additional Table 1. Code list: themes, codes, and illustrative quotes related to health-care seeking.**

| **Themes** | **Codes** | **Quotes** |
| --- | --- | --- |
| **Health-care seeking at disease onset** | **No cure only management** | “He [GP] told me himself that there is no remedy, he can give me a lot of things to alleviate the pain, but there is no remedy to take the things [complaints] away.” (Pt. 1; Female 50-65 years old, arthralgia in LE) |
|  | **Information GP duration symptoms** | “He [GP] told me you [talking in third person] will remain with the pain. […]. He told me the pain will remain inside [the joints], it does not go away easily…of course little by little it [pain] will become less.” (Pt. 13; Female 50-65 years old, arthralgia and joint stiffness in UE and LE) |
|  | **GP can only prescribe analgesics** | “You know, I am not going to complain about my leg and ankle anymore…because what will they [doctor] give me? The same painkiller, the same painkiller?” (Pt. 20; Female 50-65 years old, arthralgia and joint cramps in UE, joint stiffness in LE, and emotional distress) |
| **GPs perceptions and awareness of persistent symptoms** | **Downplaying significance/severeness symptomatology** | “I will tell him [GP] I had chikungunya and he will tell me that sometimes the symptoms will remain, you have to drink more [water], drink more. I have asked him how much can I continue to drink [confused because she is currently drinking allot of water already]?” (Pt. 12; Female 32-49 years old , arthralgia in UA and LE, and weakness in UE) |
|  | **GP awareness chronic symptoms** | “I work in the heat [in the kitchen as a cook] and the doctor [GP] told me it’s arthritis, but I did not feel that [arthritis] before I got sick [infected with chikungunya].” (Pt. 15; Female 32-49 years old, arthralgia and weakness in UE and LE) |
| **Challenges for medical referrals and support** | **Battle for referral** | “They [GPs] will give you nothing else, you [talking in third person] need to fight for yourself. […]. You need to talk for yourself to convince him [GP], you need to be able to convince him to achieve what you want to achieve [referral], if not [if you are not able to convince GP]… you can forget it [you will not get a referral].” (Pt. 7; Female 50-65 years old, arthralgia in UE and LE) |
|  | **GP: medicine instead of test/referral** | “The doctors [GPs in general] will continue giving you [talking in third person] chemical remedies. […]. I do not want remedies, I want to know why I have these symptoms.” (Pt. 1; Female 50-65 years old, arthralgia in LE) |
| **No validation of symptoms and challenges accessing therapy** | **No source of symptoms persistency** | “I even went for a test…Yes, I went to a neurologist, I went to do everything, but he [neurologist] did not see anything…I have asked him [neurologist] doctor is there anything?...because my hands are letting things fall [no grip strength] and I have cramps in my shoulder and then they [GP and neurologist] let me do the tests, but he [neurologist] did not see anything.” (Pt. 12; Female 32-49 years old, arthralgia in UE and LE, joint weakness in UE, and stiffness in LE) |
|  | **Physiotherapy increases pain** | “Yes, I have received one [referral] for massages [physiotherapy], but the massages gave me pain and the doctor [GP] stopped them…because when I received the massages, I came home with more pain.” (Pt. 6; Female >65 years old, arthralgia and joint stiffness in LE) |
|  | **Effectiveness treatment: physiotherapy** | “I am tired of going to physiotherapy. […]. It will alleviate the pain a little, but it will not cure it…there is a difference between cure and alleviation. You can’t cure it [pain] with alleviation…something will reduce and then go away, you [talking in third person] will not feel it anymore [participants definition of cure]…but this [pain] reduces and it increases again.” (Pt. 20; Female 50-65 years old, arthralgia and joint cramps in UE, joint stiffness in LE, and emotional distress) |
|  | **Out of the pocket payment physiotherapy private care** | “I went and got physiotherapy by myself at a colleague [private sector physiotherapist] eh eh wauwww, I could go to the sky [expression when something is very painful]…very painful. I cried, tears rolled from my eyes, but it helped me a lot, it was worth it. […]. That day it was painful, but I told him [private sector physiotherapist] now I am pain free…then you will notice the difference between a hand and a machine, because the machine that they currently do therapy with is like ants stinging you…and when they put it [machine] and depending how high they turn it on you will go ahhhhh [making sound of being in pain].” (Pt. 7; Female 50-65 years old, arthralgia in UE and LE) |
| **Health system restrictions** | **Limited amount of therapy sessions** | “So, the SVB [Social Insurance Bank] stopped them [physiotherapy sessions], because I had a lot of pain and for the SVB you [talking in third person] can only go a certain amount of time [sessions]…And therefore they [SVB] had to terminate me [reached amount of covered physiotherapy sessions].” (Pt 10; Female >65 years old, arthralgia in UE and LE, and joint stiffness and swelling in LE) |
|  | **HCPs are conditioned by health system** | “When I explained it [cramps] to him [GP]…they [GPs in general] are like they want to save [reduce costs on health system] something, they do not want you [talking in third person] to go to the doctor [secondary care specialist]…he [GP] will tell me no, wait a little, that sort of things…but now I will go hard on him, because he is acting like he is paying [paying participants taxes].” (Pt. 4; male 50-65 years old, joint cramps in UE and LE, and fatigue) |
| **Social stigmatization of psychological help** | **Benefits psychological help** | “I am a nurse and I know that a physical illness will always affect you emotionally and socially. A lot of people say that if you go to a psychologist you are crazy, but I know that these type of specialist can help you get out of the distress that you are in.” (Pt. 3; Female 50-65 years old, arthralgia and joint swelling in LE) |
|  | **Thoughts on psychological help participants with emotional distress** | “I do not know if it’s really necessary, I do not know. I have not received many complaints yet.” Pt. 17; Female 50-65 years old, arthralgia in UE and LE, joint locking in UE and LE, joint cramps in UE, joint swelling in LE, fatigue, and emotional distress) |
|  | **Cultural/community stigmatization** | “Again, for me I think that uhmm…not everyone is open to the guidance via a psychologist, because for us [community] when you go to a psychologist you are crazy, that is what we [community] are thinking, especially the people of older age.” (Pt. 2; Female 32-49 years old, arthralgia and joint weakness in UA and LE) |
|  | **Distrusting psychologists** | “We have a cultural barrier. […]. A lot of people in our culture are not ready to search for any [emotional] help, they think that the person [psychologist] will know their business, they [psychologist] want to know gossip…But that is not the case, you do not have to think that the person [psychologist] wants to know your gossip [emotional distress].” (Pt. 7; Female 50-65 years old, arthralgia in UE and LE) |
|  | **Psychological health seeking improvement** | “But I again believe that if the doctor [GP] takes his time and explain it to you [patient], not like we [health system] normally do with a lot of things, I will send you there, I will send you there, I will send you to that doctor [telling patients what to do instead of mutual discussion between physician and patient]. Take your time to explain the patient, you need professional [mental] help, I am not the one that can give you that guidance, you need to go there and there, you can choose yourself. But if he [GP] takes his time to explain the person [patient] I think he will win the person to go, but we need to do something about it [barriers].” (Pt. 2; Female 32-49 years old, arthralgia and joint weakness in UA and LE) |

GP = General practitioner; UE = Upper extremities; LE = Lower extremities
