# Supplementary material for: “What will the doctor give me, the same painkiller?”: a qualitative study exploring health-care seeking and symptoms self-management among patients for the treatment of long-term chikungunya disease, in Curaçao
Source: BMC Health Serv Res. 2023 Nov 13;23:1247. doi: 10.1186/s12913-023-10254-8 (PMC10641972; doi:10.1186/s12913-023-10254-8)
Supplement: Supplementary file 4 — Supplementary Material 4 [file 12913_2023_10254_MOESM4_ESM.docx]

**Additional Table 2. Code list: themes, codes, and illustrative quotes related to symptoms self-management strategies.**

| **Themes** | **Codes** | **Quotes** |
| --- | --- | --- |
| **Self-medication of symptoms** | Oral analgesics and effectiveness | “Yes, the Diclofenac, the paracetamol does not help me, that [Diclofenac] alleviates the pain much better.” (Pt. 13; Female 50-65 years old, arthralgia and joint stiffness in UE and LE) |
|  | Side effects oral analgesics | “You [talking in third person] will take a pill [analgesic] and it will alleviate the pain, but will harm your stomach. […]. Sometimes you will drink a remedy that may help you with something [complains] that you experience, but destroys something [organs] else inside the body.” (Pt. 15; Female 32-49 years old, arthralgia and weakness in UE and LE) |
|  | Reluctant taking oral analgesics | “I won’t let my body get used to pain killers. […]. Sometimes I will endure the pain, because I do not want to fill my body with pain killers.” (Pt. 13; Female 50-65 years old, arthralgia and joint stiffness in UE and LE) |
|  | Oral analgesics when pain is unbearable | “I will not keep drinking drinking [taking pain killers continually]. Some people live on those things [pain killers], I am not. I will drink one when I notice that the pain is really nagging… I will drink one.” (Pt. 7; Female 50-65 years old, arthralgia in UE and LE) |
|  | Pain management: topical analgesics | “If I am at home, I will smear something [topical analgesic] and observe how it goes. […]. I can manage [to endure the pain], because I am at home and will go nowhere…but If I need to go somewhere or to work, then I need to drink a remedy [oral analgesic].” (Pt. 17; Female 50-65 years old, arthralgia in UE and LE, joint locking in UE and LE, joint cramps in UE, joint swelling in LE, fatigue, and emotional distress) |
|  | Effectiveness topical analgesics | “I will put a little bit of gel [Diclofenac gel] for the pain. I will let my lady [girlfriend] put a little bit for me at night, before I go to sleep, but the thing [Diclofenac gel] does not work, it does not work. I have bought a lot of things [topical analgesics], but they do not work. Only the Diclofenac [oral analgesic] when you [talking in third person] drink it, it will help a little and for 2 to 3 days you will not get a lot of pain.” (Pt. 18, Male 50-65 years old, myalgia in LE) |
| **Self-management true non-pharmacological treatments** | Non-pharmacological treatment and effectiveness | “You know, there is an organisation that does certain tests, they will use a container with toxic and will let you [talking in third person] hold a device [electrodes] in it [container]…that is something that I have also done and they told me that I lack certain vitamins. I have bought the vitamins, because maybe that’s why I have it [fatigue], but they did not help [with reducing fatigue].” (Pt. 17; Female 50-65 years old, arthralgia in UE and LE, joint locking in UE and LE, joint cramps in UE, joint swelling in LE, fatigue, and emotional distress) |
|  | Non-pharmacological management and effectiveness | “I will lay down flat [on the ground], lay down flat and really take my time, after a while it [pain] will reduce. It will not go away completely completely, but it [pain] just reduces a little.” (Pt. 16; Female 32-49 years old, arthralgia and joint stiffness in UE and LE) |
|  | Natural remedies and effectiveness | “I believe a lot in lemons, lemons really help, especially for symptoms that are rheumatic. I have drunk a lot of lemons and it helps cleaning the joints and blood…and I can say that relatively the swelling is less compared to the beginning [acute disease]. Here [pointing at lower leg] it is less, if I can say that it’s slimmer because of the lemons, I do not know.” (Pt. 3; Female 50-65 years old, arthralgia and joint swelling in LE) |

UE = Upper extremities; LE = Lower extremities
